# Cholera Inoculation

**Published:** 1916-05

**Authors:** 


**Cholera Inoculation.** Buiwid and Arzt, Wien. Med. Woch. report 47 cases with 4 deaths, all four having had but a single injection whereas three are considered necessary to protect.

